# Supplementary material for: Modulatory Effects of Atractylodin and β-Eudesmol on Human Cytochrome P450 Enzymes: Potential Drug-Drug Interactions
Source: Molecules. 2023 Mar 31;28(7):3140. doi: 10.3390/molecules28073140 (PMC10095747; doi:10.3390/molecules28073140)
Supplement: Supplementary file 1 [file molecules-28-03140-s001.zip › molecules-2310064-supplementary.pdf]

Supplementary Information

Modulatory Effects of Atractylodin and β-Eudesmol on Human Cytochrome P450 Enzymes: Potential Drug-Drug Interactions

Artitaya Thiengsusuk [1], Tullayakorn Plengsuriyakarn [1,2] and Kesara Na-Bangchang [1,2,*]

[1] Graduate Studies, Chulabhorn International College of Medicine, Thammasat University, Pathumthani 12120, Thailand
[2] Center of Excellence in Pharmacology and Molecular Biology of Malaria and Cholangiocarcinoma, Thammasat University, Pathumthani 12120, Thailand
[3] Drug Discovery and Development Center, Office of Advanced Science and Technology, Thammasat University, Pathumthani 12120, Thailand
* Correspondence: kesaratmu@yahoo.com

Table S1. The fold-changes of mCYP1A2 and mCYP3A11 mRNA expression levels in mouse livers.

Fold-changes of mCYP1A2 mRNA levels Median (95% CI)				Fold-changes of mCYP3A11 mRNA levels Median (95% CI)			
**Exposure period**	**Control**	**Atactylodin**	**β-eudesmol**	**Exposure period**	**Control**	**Atactylodin**	**β-eudesmol**
**1 day**	1 (1-1)	1.60 (1.28-2.14)*	2.63 (2.21-3.43)*	**1 day**	1 (1-1)	1.72 (1.03-2.22)*	2.18 (1.97-2.38)*
**7 days**	1 (1-1)	1.20 (0.93-1.33)**	1.44 (0.97-1.49)**	**7 days**	1 (1-1)	1.3 (0.82-1.65)	1.50 (1.05-1.79)*
**14 days**	1 (1-1)	0.89 (0.76-0.97)*	1.37 (1.21-1.92)*	**14 days**	1 (1-1)	0.87 (0.58-1.12)**	0.96 (0.59-1.32)
**21 days**	1 (1-1)	0.71 (0.45-0.83)*	0.91 (0.73-0.99)*	**21 days**	1 (1-1)	0.62 (0.48-0.64)*	0.78 (0.38-0.95)*

Data are expressed as median (95% CI) from three experiments (duplicate each). Statistical significance with *p = 0.002, **p = 0.04 compared with control.

Table S2. Protein expression ratios of mCYP1A2 and mCYP3A11 in mouse livers.

	Protein expression ratio of mCYP1A2 Median (95% CI)			Protein expression ratio of mCYP3A11 Median (95% CI)		
**Exposure period**	**Control**	**Atactylodin**	**β-eudesmol**	**Control**	**Atactylodin**	**β-eudesmol**
**1 day**	1.5 (1.32-1.60)	1.39 (1.19-1.70)	1.06 (0.70-1.20)**	1.73 (1.37-1.88)	1.82 (1.55-2.14)	1.50 (1.29-1.72)
**7 days**	1.88 (1.60-2.10)	1.55 (1.30-1.74)	1.83 (1.40-1.94)	1.57 (1.38-2.18)	1.26 (1.21-1.79)*	1.62 (1.29-1.70)
**14 days**	2.42 (1.70-2.72)	1.77 (1.70-2.00)	2.05 (1.87-2.70)	2.31 (1.90-2.63)	1.67 (1.39-1.74) **	1.60 (1.36-1.70) **
**21 days**	2.20 (2.18-2.42)	1.33 (0.90-1.45)**	1.00 (0.67-1.10) **	2.18 (1.98-2.61)	1.41 (1.20-1.77) **	1.42 (1.23-1.79) **

The data are expressed as median (95% CI) from six replications. Statistical significance with *p = 0.039, **p = 0.004 compared with control.

Table S3. The mCYP1A2- and mCYP3A11-mediated metabolites (paracetamol and dehydronifedipine) in mouse liver microsomes.

	Paracetamol concentration (μM) Median (95% CI)			Dehydronifedipine concentration (μM) Median (95% CI)		
**Exposure period**	**Control**	**Atactylodin**	**β-eudesmol**	**Control**	**Atactylodin**	**β-eudesmol**
**1 Day**	11.40 (10.70-13.70)	15.80 (13.00-18.20)**	12.35 (11.50-14.80)	6.70 (5.80-8.20)	5.35 (4.80-6.00)***	6.90 (5.10-7.70)
**7 Days**	12.25 (10.70-13.50)	12.00 (9.30-13.40)	11.80 (11.20-13.30)	6.90 (6.00-8.20)	5.20 (4.40-5.70)*	7.5 (5.50-8.00)
**14 Days**	12.80 (11.00-16.30)	14.50 (12.00-15.20)	13.25 (11.70-13.80)	7.55 (6.30-7.80)	6.10 (4.90-7.20)****	4.00 (3.10-4.40)*
**21 Days**	12.90 (11.20-15.30)	10.60 (8.70-11.90)***	8.30 (7.50-9.40)*	7.75 (6.70-8.40)	3.75 (3.30-4.00)*	3.20 (2.70-3.50)*

The data are expressed as median (95% CI) from three experiments (duplicate each). Statistical significance with *p = 0.004, **p = 0.006, ***p = 0.010, ****p = 0.016 compared with control.

Table S4. Specific forward and reverse primer sequences used for the analysis of mRNA expressions of mCYP1A2 and mCYP3A11.

Gene	Primer sequence
mCYP1A2	Forward: 5′-TGGTGGAATCGGTGGCTAAC-3′<br>Reverse: 5′- GACCGGGAAGAAGTCCACTG-3′
mCYP3A11	Forward: 5′- ACCTGGGTGCTCCTAGCAAT-3′<br>Reverse: 5′- GCACAGTGCCTAAAAATGGCA-3′
β-actin	Forward 5′- AACCCTAAGGCCAACCGTGAAAAG-3′<br>Reverse 5′- CGACCAGAGGCATACAGGGACAAC-3′

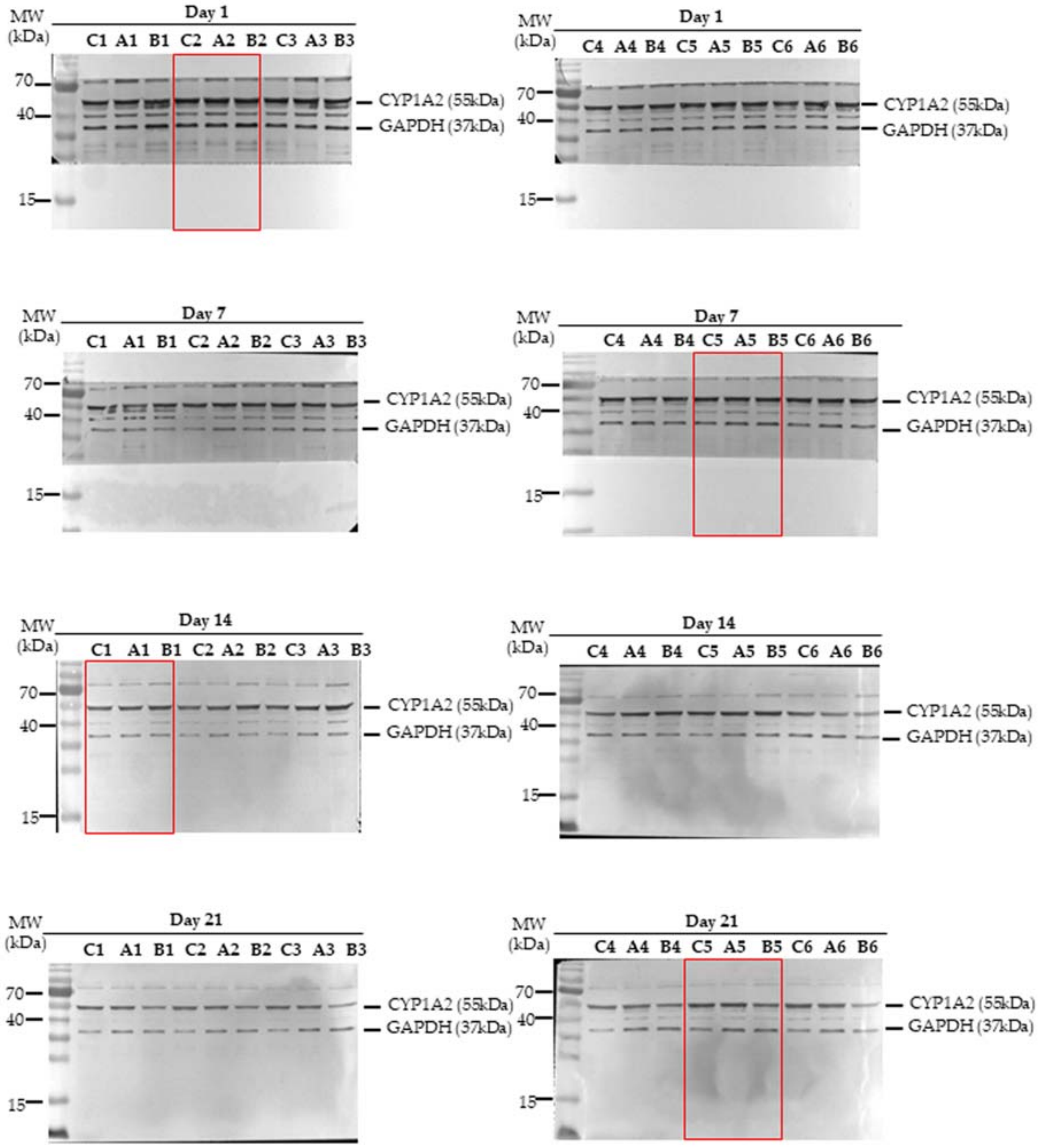

Figure S1. RAW Images. Images of the mCYP1A2 western blot immunoblot analysis performed in the livers of male ICR mice treated for 1, 7, 14, and 21 days with 100 mg/kg BW atractylodin or β-eudesmol (red lanes in the box were cropped and shown in Fig. 2B).

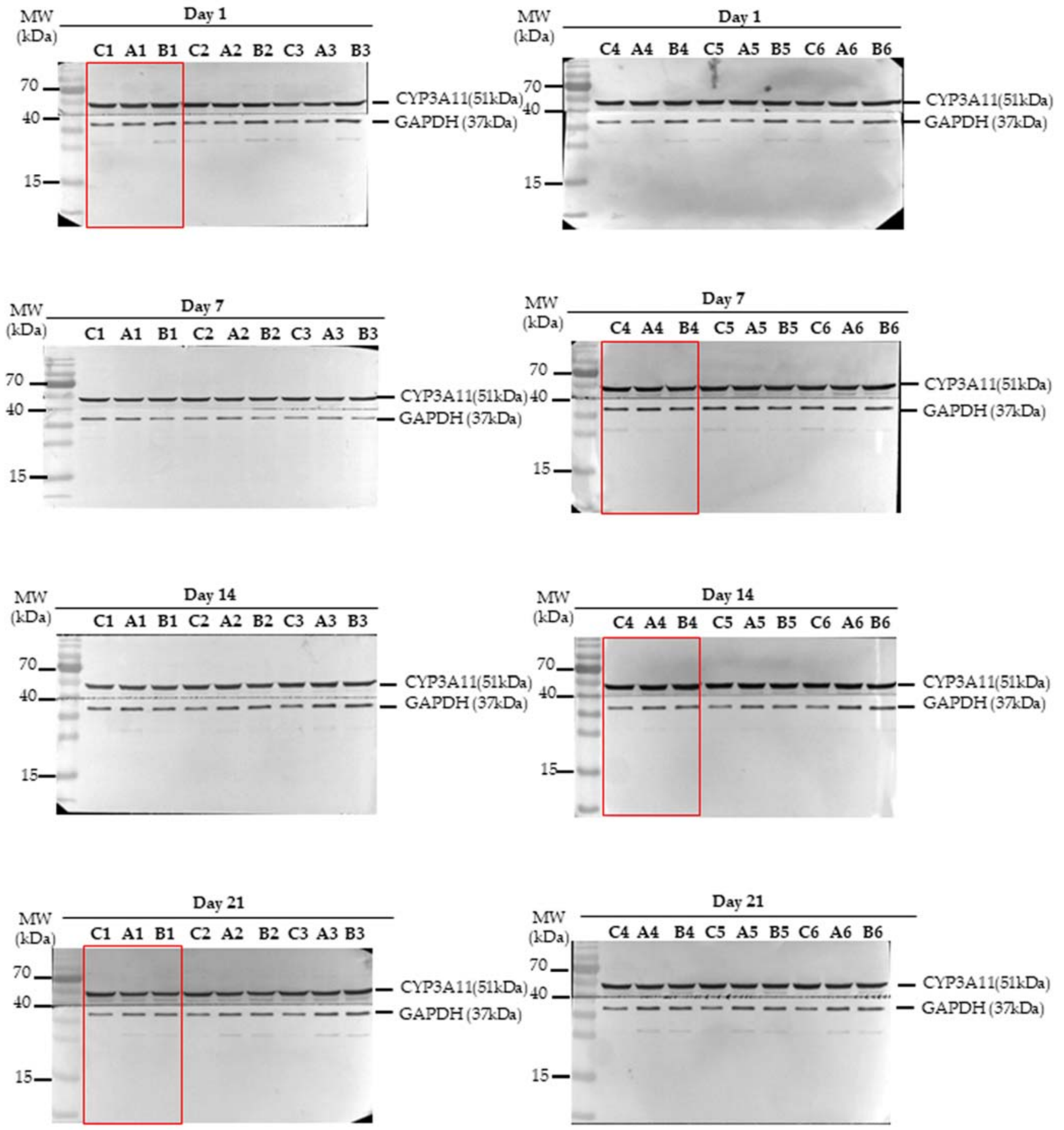

Figure S2. RAW Images. Images of the mCYP3A11 western blot immunoblot analysis performed in the livers of male ICR mice treated for 1, 7, 14, and 21 days with 100 mg/kg BW atractylodin or β-eudesmol (red lanes in the box were cropped and shown in Fig. 2D).
